# The Importance of Health Insurance in Addressing Asian American Disparities in Utilization of Clinical Preventive Services: 12-Year Pooled Data from California

**DOI:** 10.1089/heq.2020.0008

**Published:** 2020-07-02

**Authors:** Sara B. McMenamin, Nadereh Pourat, Richard Lee, Nancy Breen

**Affiliations:** ^1^ Department of Family Medicine and Public Health, University of California, San Diego, La Jolla, California, USA.; ^2^ Department of Health Policy and Management, Fielding School of Public Health, University of California, Los Angeles, Los Angeles, California, USA.; ^3^ Information Management Services, Inc., Rockville, Maryland, USA.; ^4^ National Cancer Institute, NIH, Rockville, Maryland, USA.

**Keywords:** health disparities, Asian Americans, health insurance, preventive services

## Abstract

**Purpose:** Previous research has shown that Asian Americans are less likely to receive recommended clinical preventive services especially for cancer compared with non-Hispanic whites. Health insurance expansion has been recommended as a way to increase use of these preventive services. This study examines the extent to which utilization of preventive services by Asians overall and by ethnicity compared with non-Hispanic whites is moderated by health insurance.

**Methods:** Data from the California Health Interview Survey (CHIS) was used to examine preventive service utilization among non-Hispanic whites, Asians, and Asian subgroups 50–64 years of age by insurance status. Six waves of CHIS data from 2001 to 2011 were combined to allow analysis of Asian subgroups. Logistic regression models were run to predict the effect of insurance on receipt of mammography, colorectal cancer (CRC) screening, and flu shots among Asians overall and by ethnicity compared with whites.

**Results:** Privately insured Asians reported significantly lower adjusted rates of mammography (83.1% vs. 87.6%) and CRC screening (54.7% vs. 59.4%), and higher rates of influenza vaccination (48.7% vs. 38.5%) than privately insured non-Hispanic whites. Adjusted rates of cancer screening were lower among Koreans and Chinese for mammography, and lower among Filipinos for CRC screening.

**Conclusion:** This study highlights the limitations of providing insurance coverage as a strategy to eliminate disparities for cancer screening among Asians without addressing cultural factors.

## Introduction

With the release of the 2003 Institute of Medicine report, *Unequal Treatment: Confronting Racial and Ethnic Disparities in Health Care*, reducing health disparities was underscored as a national priority in the United States.^1^ Reducing racial/ethnic gaps in clinical preventive screening rates recommended by the United States Preventive Services Task Force (USPSTF) is a critical component of reducing overall health disparities.^2^

Previous research has documented disparities in the receipt of clinical preventive services by race/ethnicity.^3^ This research has found that Asian Americans are less likely to receive recommended clinical preventive services related to cancer compared with non-Hispanic whites.^3–8^ In addition, Asian Americans have lower screening rates for certain cancers compared with blacks and Hispanics despite having a higher socioeconomic status compared with these groups.^9^

Studies of Asian Americans are largely limited to aggregate analyses of this diverse population because the sample size for Asian subgroups in most surveys is too small for in-depth analyses. Because the proportion of Asians is much higher in California than in the United States,^10^ and because the overall sample size is large, the California Health Interview Survey (CHIS) is a unique ongoing data source for studying disparities between Asian American subgroups. Subsequent research has reported on use of clinical preventive services among Asian subgroups, with most studies conducted in community clinics serving one Asian subgroup that does not allow for comparison.^11–14^

Previous studies using CHIS compared utilization patterns of Asians by ethnicity and found wide variation in utilization patterns in colorectal and breast cancer screening.^6,8,9,15,16^ Consistent findings of variations in characteristics, insurance status, and outcomes among Asian American subgroups have led to continued recommendations for disaggregated analyses of Asian Americans by ethnicity and/or country of origin.^17^

The association between health insurance coverage and the use of clinical preventive services is well established in the general population.^18,19^ How insurance coverage contributes to the use of preventive services among Asian American ethnicities is less well understood. Previous research found that, even after adjusting for insurance status, Asians had lower rates of receipt of colorectal screening.^6,15^ Studies examining cancer screening among Asian women by health insurance status found that the impact of health insurance status on receipt of cancer screening varied significantly by subgroup.^7,16,20^ Even though cancer screening rates are lower among Asians, studies show that the human papilloma virus vaccine uptake tends to be higher among Asians than among non-Hispanic whites, suggesting the possibility of a greater cultural acceptance of vaccinations to prevent cancer compared with cancer screening among Asian populations.^21,22^

This study explores why Asians have lower cancer screening rates than whites, even when insured. We examine data spanning 12 years to assess the potential role of insurance status on the utilization of three different clinical preventive services, two cancer screening modalities, and one type of vaccination (i.e., mammography, colorectal cancer (CRC) screening, and flu shots). These preventive services were selected from the list of preventive services covered under the Affordable Care Act (ACA) that were included in five or more sequential versions of CHIS and are recommended for the adult population of interest (i.e., adults 50–64 years of age). We hypothesize that having insurance will increase the likelihood of the use of preventive services but with less potency for Asian Americans than for non-Hispanic whites, especially for cancer screening services. Moreover, we anticipate that potency will vary by Asian ethnicity.

## Methods

### Data

Data are from CHIS, a population-based telephone survey conducted every other year since 2001 and continuously beginning in 2011 on a representative sample of 42,000–56,000 persons in California households. Data for this study combined six waves of the survey conducted in 2001, 2003, 2005, 2007, 2009, and 2011. Data were available for the following measures and survey waves: mammography (2001–2011), CRC screening (2001–2009), and flu shot (2003–2011). Items were not administered for these three preventive services in the 2013 cycle, so uninterrupted long-term trends in screening could not be examined in subsequent survey years. The CHIS oversampled Asian subgroups such as Vietnamese and Korean during select years. Interviews were conducted in Mandarin, Cantonese, Korean, and Vietnamese to include Asian respondents with limited or no English proficiency. Studies using CHIS have IRB approval through UCLA (no. 17-000362).

The study population was limited to non-Hispanic whites (*n*=55,599) and non-Hispanic Asians (*n*=6526) ages 50–64. This age group was selected for three reasons. First, the USPSTF clinical practice guidelines recommend screening for both mammography and CRC for ages 50–64 throughout the study period.^23,24^ Second, persons over 64 were excluded because they are largely covered by Medicare, and thus not at risk for fluctuations in health insurance coverage. Finally, the 50–64 age group was singled out by the Centers for Disease Control and Prevention (CDC), AARP, and the American Medical Association (AMA) as an important target audience for improving screening rates.^25^

### Measures

#### Outcome variables

Three dichotomous outcomes are used to measure clinical preventive services. The USPSTF recommends screening for breast cancer using mammography every 2 years for women ages 50 and older;^26^ and for CRC beginning for adults age 50 and older.^27^ The Advisory Committee on Immunization Practices recommends annual vaccination for influenza (i.e., receipt of the flu shot) for everyone over 6 months of age.^28^ Variables were coded a value of “1” for respondents who had the service within the recommended time interval and “0” for those who did not.

#### Independent variables of interest

Interactions by race/ethnicity, Asian subgroup, and insurance status are the main independent variables of interest. Race and ethnicity variables were combined to examine non-Hispanic Asian Americans (Asian) vs. non-Hispanic whites (white). Asians were further categorized by ethnicity into Chinese, Filipino, Japanese, Korean, South Asian, Vietnamese, and Other Asian. Insurance was categorized as public only, private, or uninsured. Private insurance includes insurance purchased by individuals or employers. Respondents without public or private insurance were classified as uninsured. To compare the role of insurance among Asians and whites, six variables were created to capture the interaction between race and insurance status: Asian-private insurance, Asian-public insurance, Asian-uninsured, white-private insurance, white-public insurance, and white-uninsured. Similarly, 24 variables were created to capture the interaction of insurance and ethnicity among Asian subgroups.

#### Control variables

The Andersen Behavioral Model of Health Services Use was used as a framework to select other determinants of health care use such as predisposing, enabling, or need factors.^29^ *Predisposing factors* included gender, marital status (married vs. single), educational attainment (less than high school, some college, college graduate, higher than college graduate), and employment status (employed vs. unemployed). English language proficiency (native English speaker, speaks very well/well vs. not well/not at all), and percent of lifetime spent in the United States (0–49%, 50–99%, 100%) were used to measure level of acculturation. These two measures of acculturation along with federal poverty level (<100%, 100% to <200%, and 200% or higher) and having a “usual place to go when sick or needing care” (as defined by the respondent) were *enabling factors*. The *need* for health care was measured by variables addressing health status, including self-assessed general health status (excellent/very good/good vs. fair/poor) and smoking status (current smoker, not current smoker). Age of respondent and year of survey were included in the regression models to account for variations in service use over time with the 2011 cycle of CHIS as the reference group for mammogram and flu shot and 2009 as the reference group for CRC screening.

### Statistical analyses

Two weighted logistic regression models were run to predict the effect of insurance on receipt of mammography, CRC screening, and flu shots among Asians as a group (model 1) and by ethnicity (model 2) compared with whites. These two models were run three times on each outcome with different referent groups (white-private; white-public, white-uninsured) to compare different racial/ethnic and insurance categories. The same control variables were included in both models. Cell sizes were examined to ensure a sufficient number of respondents for stable estimates. Tables showing the white-private referent group are provided, and significant differences between racial/ethnic and insurance categories are discussed in the text. *p*-values for multiple comparisons were adjusted using the Bonferroni correction; however, results are presented without the adjustment to avoid Type II error.

Predicted probabilities (marginals) are computed from the weighted logistic regression models using the “PREDMARG” statement of the logistic procedure in SUDAAN. Predicted probabilities are estimated for every variable in the model while controlling for each covariate in the model.^30,31^ Predicted probabilities allow direct comparison of utilization among groups. Predicted probabilities and their 95% confidence intervals are presented in the tables. Full logistic regression models with odds ratios are presented in the Appendix Tables A1 and A2. Analyses were conducted in 2019–2020 using SAS 9.4, SAS Institute, Cary, NC and SUDAAN 11.0.1, RTI International, Research Triangle Park, NC. Models were weighted to account for the complex sample design of CHIS.

## Results

White and Asian Californians 50–64 years of age differed significantly in their characteristics (Table 1). In terms of *predisposing* characteristics, Asians had a higher percentage of females, had lower levels of postcollege education and lower incomes, and were more likely to be married or living as married compared with whites. In terms of *enabling* characteristics, Asians also had lower levels of acculturation with only 67% reporting proficiency in English (compared with 99.6% of whites) and 87% reporting being born outside of the United States compared with 8% of whites. There were significant differences in type of health insurance coverage, with whites being more likely to have private coverage (83.3% vs. 73.3%), less likely to be uninsured (8.4% vs. 15.6%), and more likely to have a usual place to go for health care (92.5% vs. 87.9%). In terms of *need* for health care, whites had higher rates of daily smoking but reported better overall health status than Asians.

**Table 1.** Characteristics of Non-Hispanic White and Asian Adults 50–64 in California, 2001–2011

	Non-Hispanic white		Non-Hispanic Asian	
	*n*	% (95% CI)	*n*	% (95% CI)
Gender				
Male	23,142	**48.5 (48.0–49.0)**	2822	**44.4 (42.7–46.1)**
Female	32,457	**51.5 (51.0–52.0)**	3704	**55.6 (53.9–57.3)**
Marital status				
Married or living as married	34,354	**72.7 (72.2–73.3)**	4980	**82.6 (81.2–84.0)**
Other	21,215	**27.3 (26.7–27.8)**	1543	**17.4 (16.0–18.8)**
Education				
High school or less	11,640	**24.3 (23.7–24.8)**	2031	**32.2 (30.4–34.0)**
Some college	17,039	**29.1 (28.5–29.6)**	1165	**17.1 (15.7–18.6)**
College graduate	13,801	**24.1 (23.6–24.6)**	2099	**33.5 (31.8–35.4)**
More than college	13,119	**22.6 (22.1–23.0)**	1231	**17.2 (15.9–18.5)**
Employment status				
Employed	37,636	69.8 (69.2–70.4)	4369	70.5 (68.8–72.1)
Unemployed	17,869	30.2 (29.6–30.8)	2147	29.5 (27.9–31.2)
English proficiency				
English proficient	55,452	**99.6 (99.5–99.7)**	4098	**67.0 (65.2–68.7)**
Limited or no English proficiency	145	**0.4 (0.3–0.5)**	2428	**33.0 (31.3–34.8)**
Percent of time in U.S.				
0–49	1188	**2.6 (2.2–3.0)**	3376	**52.1 (50.4–53.8)**
50–99	2624	**5.4 (5.1–5.8)**	2138	**34.4 (32.8–36.1)**
100	51,786	**92.0 (91.6–92.3)**	1008	**13.5 (12.4–14.6)**
Income (percent of FPL)				
<100 FPL	2974	**4.5 (4.2–4.8)**	996	**12.0 (10.9–13.2)**
100–<200 FPL	5290	**8.3 (7.9–8.6)**	1084	**16.8 (15.4–18.2)**
200+ FPL	47,335	**87.2 (86.7–87.7)**	4446	**71.2 (69.5–72.9)**
Health insurance coverage				
Any private	45,255	**83.3 (82.8–83.8)**	4531	**73.3 (71.7–74.9)**
Public only	5531	**8.3 (7.9–8.7)**	868	**11.1 (10.0–12.3)**
Uninsured	4813	**8.4 (8.0–8.9)**	1127	**15.6 (14.4–16.8)**
Have usual place to go when need care sick or needing health advice				
Yes	51,396	**92.5 (92.1–92.9)**	5658	**87.9 (86.6–89.0)**
No or ER is usual source	4188	**7.5 (7.1–7.9)**	868	**12.1 (11.0–13.4)**
General health status				
Excellent/very good/good	47,147	**85.4 (84.9–85.9)**	4434	**71.9 (70.2–73.5)**
Fair/poor	8445	**14.6 (14.1–15.1)**	2091	**28.1 (26.5–29.8)**
Smoking status				
Current smoker	8371	**15.1 (14.6–15.6)**	655	**10.4 (9.3–11.6)**
Not a current smoker	47,220	**84.9 (84.4–85.4)**	5869	**89.6 (88.4–90.7)**

Source: California Health Interview Survey 2001, 2003, 2005, 2007, 2009, 2011/2012.

In cases where there are missing responses, the total of the categories will not equal the total sample size (i.e., *n*=55,599 for non-Hispanic white and *n*=6526 for non-Hispanic Asian).

Boldface indicates a statistically significant difference (*p*≤0.05) in rates between non-Hispanic whites and non-Hispanic Asians.

CI, confidence interval; CHIS, California Health Interview Survey; ER, emergency room.

Across all insurance types, whites had higher rates of mammography and CRC screening while Asians reported higher flu shot rates (Table 2). The same pattern was seen among the privately insured, with whites having higher rates of mammography and CRC screening, but lower rates of flu shots than Asians. Among the publicly insured, there was no difference in mammography or flu shot rates, but there was a lower rate of CRC screening among Asians. Among the uninsured, the only significant difference, was a higher rate of flu shots for Asians compared with whites.

**Table 2.** Preventive Screening Rates by Health Insurance Coverage for Non-Hispanic Whites and Non-Hispanic Asians, Ages 50–64, CHIS 2001–2011

	Mammography^a^ (2001–2011)	CRC screening^b^ (2001–2009)	Flu shot^c^ (2003–2011)
	% (95% CI)	% (95% CI)	% (95% CI)
All insurance types			
Non-Hispanic white	**85.5 (84.8–86.1)**	**58.6 (57.9–59.3)**	**39.0 (38.3–39.6)**
Non-Hispanic Asian	**79.9 (77.8–81.8)**	**48.1 (46.0–50.2)**	**42.9 (41.0–44.9)**
Any private insurance^d^			
Non-Hispanic white	**88.9 (88.4–89.5)**	**61.6 (60.9–62.3)**	**40.3 (39.6–41.0)**
Non-Hispanic Asian	**85.1 (82.7–87.2**)	**53.1 (50.6–55.6)**	**44.9 (42.7–47.2)**
Public insurance^e^			
Non-Hispanic white	78.0 (75.2–80.5)	**56.9 (54.1–59.6)**	44.7 (42.2–47.2)
Non-Hispanic Asian	80.5 (73.8–85.9)	**43.4 (37.4–49.6)**	48.5 (42.5–54.5)
Uninsured^f^			
Non-Hispanic white	55.8 (52.1–59.3)	27.9 (24.9–31.1)	**20.3 (17.6–23.2)**
Non-Hispanic Asian	54.3 (48.6–59.9)	27.1 (23.4–31.3)	**28.8 (24.7–33.2)**

Source: CHIS 2001, 2003, 2005, 2007, 2009, 2011/2012.

Boldface indicates a statistically significant difference (*p*≤0.05) in rates between Non-Hispanic white and Non-Hispanic Asian.

^a^ Mammogram within the past 2 years.

^b^ FOBT in past year, sigmoidoscopy in the past 5 years, or colonoscopy within the past 10 years.

^c^ Flu shot within the past year.

^d^ Private insurance includes insurance purchased by individuals or employers.

^e^ Public insurance includes Medicare, Medicaid, and other forms of public coverage such as military.

^f^ Anyone without public or private insurance was classified as uninsured.

CRC, colorectal cancer; FOBT, fecal occult blood test.

Table 3 presents the predicted probabilities of preventive service utilization by insurance type and race, based on the logistic regression models. Asians with private health insurance had significantly lower probability of mammography screening (83.1% vs. 87.6%), CRC screening (54.7% vs. 59.4%), and higher probability of flu shots (48.7% vs. 38.5%) compared with whites with private health insurance. Among the publicly insured, Asians had a higher predicted probability of flu shots compared with whites; differences for mammography and CRC screening were not significant. Among the uninsured, the only significant difference was that Asians had higher rates of flu shots compared with whites.

**Table 3.** Predicted Probabilities^a^ of Mammography, Colorectal Cancer Screening, and Flu Shot by Insurance Type and Race, Ages 50–64

	Mammography^b^ (2001–2011)	CRC screening^c^ (2001–2009)	Flu shot^d^ (2003–2011)
	Predicted probabilities (95% CI)	Predicted probabilities (95% CI)	Predicted probabilities (95% CI)
Private insurance^e^			
White (referent)	87.6 (86.9–88.4)	59.4 (58.6–60.3)	38.5 (37.8–39.3)
Asian	83.1 (80.2–85.6)	54.7 (52.0–57.4)	48.7 (46.0–51.4)
Public insurance^f^			
White (referent)	84.1 (81.6–86.3)	59.4 (56.7–62.0)	43.0 (40.3–45.9)
Asian	85.3 (78.8–90.1)	53.2 (46.4–59.9)	55.5 (48.7–62.0)
Uninsured^g^			
White (referent)	68.0 (64.8–71.0)	36.7 (33.4–40.1)	**25.5 (22.4–29.0)**
Asian	64.4 (57.1–71.2)	40.3 (35.0–46.0)	**40.5 (34.6–46.6)**

Source: CHIS 2001, 2003, 2005, 2007, 2009, 2011/2012.

Boldface indicates a statistically significant difference (*p*≤0.05) in rates between Non-Hispanic white with private insurance (i.e., the referent group).

These models were adjusted for gender, marital status, education, employment, English language proficiency, percent of lifetime spent in the United States, income, having a usual place to go when sick or needing care, general health status, smoking status, age, and year of survey. The full models can be found in Appendix Table A1.

^a^ The predicted probabilities (marginals) are adjusted rates derived from logistic regression models.

^b^ Mammogram within the past 2 years.

^c^ FOBT in the past year, sigmoidoscopy in the past 5 years, or colonoscopy within the past 10 years.

^d^ Flu shot within the past year.

^e^ Private insurance includes insurance purchased by individuals or employers.

^f^ Public insurance includes Medicare, Medicaid, and other forms of public coverage such as military.

^g^ Anyone without public or private insurance was classified as uninsured.

Table 4 compares predicted probabilities for use of preventive services by insurance type for six Asian ethnicities. The total of 6526 Asian respondents is composed of the following: 1961 Chinese (30%), 1345 Vietnamese (21%), 937 Filipino (14%), 879 Korean (13%), 651 Japanese (10%), 422 South Asian (6%), 323 other Asian (5%), and 8 respondents with unknown ethnicity. Data were not shown separately for other Asian or unknown Asian ethnicity due to their small sample sizes. Among Asian Americans with private insurance, there were lower predicted probabilities of mammography screening among Koreans (75.5%) and Chinese (80.2%) compared with whites with private insurance (87.6%). There was a lower predicted probability of colorectal screening among Filipinos with private insurance (51.1%) compared with whites (59.3%). All privately insured Asian American subgroups, except for South Asian, had a higher predicted probability of flu shots compared with privately insured whites.

**Table 4.** Predicted Probabilities^a^ of Mammography, Colorectal Cancer, and Flu Shot by Insurance Type and Asian Ethnicity, Ages 50–64

Race and insurance type	Mammography^b^ (2001–2011)	CRC^c^ (2001–2009)	Flu^d^ (2003–2011)
	Predicted probabilities (95% CI)	Predicted probabilities (95% CI)	Predicted probabilities (95% CI)
Private insurance^e^			
White (referent)	87.6 (86.8–88.3)	59.3 (58.5–60.2)	38.3 (37.5–39.1)
Chinese	**80.2 (75.7–84.1)**	58.1 (54.3–61.9)	**46.1 (42.4–49.8)**
Filipino	84.6 (78.5–89.2)	**51.0 (45.3–56.8)**	**50.3 (44.4–56.2)**
Japanese	85.5 (79.8–89.7)	55.0 (49.3–60.6)	**47.9 (42.3–53.6)**
Korean	**75.5 (65.2–83.5)**	51.9 (44.8–58.9)	**54.3 (46.6–61.8)**
South Asian	81.7 (73.4–87.9)	50.2 (41.7–58.7)	45.7 (39.1–52.4)
Vietnamese	88.5 (79.4–93.9)	64.9 (57.9–71.3)	**57.5 (49.4–65.3)**
Public insurance^f^			
White (referent)	84.4 (82.0–86.6)	59.5 (56.8–62.1)	43.1 (40.3–45.9)
Chinese	80.9 (60.7–92.1)	50.8 (37.0–64.6)	51.0 (37.4–64.4)
Filipino	84.9 (69.5–93.3)	69.7 (56.1–80.6)	49.9 (35.8–63.9)
Japanese	68.0 (27.2–92.3)	31.5 (11.4–62.2)	45.7 (22.6–70.8)
Korean	82.6 (64.5–92.5)	**38.5 (22.8–57.0)**	**73.2 (56.2–85.3)**
South Asian	59.8 (16.2–92.0)	32.5 (6.6–76.7)	27.2 (8.7–59.4)
Vietnamese	88.8 (80.1–94.0)	56.1 (47.5–64.4)	**63.0 (53.6–71.6)**
Uninsured^g^			
White (referent)	68.1 (64.9–71.2)	36.7 (33.3–40.1)	25.5 (22.4–28.9)
Chinese	64.7 (52.5–75.3)	32.9 (24.9–41.9)	34.6 (26.4–43.9)
Filipino	64.0 (44.7–79.7)	42.1 (30.1–55.2)	41.6 (24.7–60.8)
Japanese	**37.9 (20.2–59.5)**	33.7 (16.7–56.3)	26.2 (11.0–50.5)
Korean	**54.8 (43.1–66.0)**	33.9 (25.6–43.2)	**47.1 (37.3–57.1)**
South Asian	66.9 (30.9–90.1)	32.5 (12.4–62.1)	36.7 (14.5–66.5)
Vietnamese	**80.4 (70.2–87.7)**	**60.5 (49.1–70.9)**	**54.3 (43.9–64.3)**

Source: CHIS 2001, 2003, 2005, 2007, 2009, 2011/2012.

Boldface indicates a statistically significant difference (*p*≤0.05) in rates between Non-Hispanic white (i.e., the referent group).

These models were adjusted for gender, marital status, education, employment, English language proficiency, percent of lifetime spent in the United States, income, having a usual place to go when sick or needing care, general health status, smoking status, age, and year of survey. The full models can be found in Appendix Table A2.

Adjusted for demographic, acculturation, and health care status and access characteristics.

^a^ The predicted probabilities (marginals) are adjusted rates derived from logistic regression models.

^b^ Mammogram within the past 2 years.

^c^ FOBT in the past year, sigmoidoscopy in the past 5 years, or colonoscopy within the past 10 years.

^d^ Flushot within the past year.

^e^ Private insurance includes insurance purchased by individuals or employers.

^f^ Public insurance includes Medicare, Medicaid, and other forms of public coverage such as military.

^g^ Anyone without public or private insurance was classified as uninsured.

Analyses of Asian American ethnicities with public insurance revealed slightly different patterns in preventive services use. Only Koreans with public insurance had a significantly lower predicted probability of CRC screening compared with whites with public insurance (38.2% vs. 59.5%). Koreans (73.0%) and Vietnamese (62.9%) had significantly higher predicted probabilities of flu shots compared with whites with public insurance (43.1%).

Analysis comparing uninsured Asians and whites showed that Japanese (38.1%) and Koreans (54.5%) had lower predicted probability of mammogram compared with uninsured whites (68.1%). Finally, uninsured Vietnamese had higher predicted probability of utilization for all three preventive services compared with uninsured whites: mammogram (80.1% vs. 68.1%), CRC screening (60.1% vs. 36.6%), mammograms, and flu shots (54.0% vs. 25.5%).

Odds ratios for the year of survey in the models showed that use of mammography and flu shots did not increase over time (Appendix Tables A1 and A2). In contrast, use of CRC screening did increase over time.

## Discussion

Three new findings emerged from this study: (1) disparities in use of preventive services among Asians and whites occur primarily within the privately insured population; (2) Asians across all insurance types are more likely to get flu shots, and (3) uninsured Vietnamese Americans had higher rates of all three preventive services compared with uninsured whites. The implications of each finding is discussed below.

First, our results showed that disparities in mammography and CRC screening occurred primarily among the privately insured population. The ACA was successful in increasing insurance coverage and reducing the disparity in insurance rates between Asians and whites, especially for Koreans and Vietnamese, primarily through increases in private insurance coverage.^33,34^ A recent analysis found that despite increases in private insurance coverage, disparities in utilization of and access to health care services between Asians and whites remained.^35,36^ More research is needed to understand why Asians with private insurance are less likely to utilize cancer screenings compared with whites–but not among those with public insurance or uninsured. It is possible that campaigns to increase preventive services among Asians have focused on publicly insured and uninsured Asian Americans. This may be one area ripe for outreach by health educators in private health insurance plans. Continuing medical education for Asian physicians is another strategy that has worked to increase cancer screening.^37^

Second, across all three types of insurance status categories, Asians were more likely to report receiving a flu shot compared with whites. Previous research has suggested that Asians have lower use of screening modalities and higher use of vaccinations such as flu shots.^16,20^ It is unclear if lower mammography and colorectal screenings among Asians is due to the complexity or invasiveness of the modality or to access barriers related to having to go outside of the primary care setting to obtain screening services.^38^ To increase use of such cancer screenings among insured Asians, qualitative research to better understand perceived barriers, knowledge gaps, language barriers, and other concerns is warranted.^39^

Finally, our results show that uninsured Vietnamese Americans had higher rates of utilization of all three clinical preventive services compared with uninsured whites. Sustained targeted outreach to the Vietnamese community in Northern California since 1986 through the Vietnamese Community Health Promotion Project (VCHPP) likely accounts for these higher rates.^37,39–41^ By targeting the range of diseases prevalent among Vietnamese, and remaining largely focused on the local Vietnamese community over decades, the VCHPP built trust in the community, including among doctors and researchers. VDHPP conducts continuing medical education for Vietnamese physicians, hires lay health workers, and recruits volunteers for research studies. Similar consistent health promotion interventions could be used to increase screening rates among other Asian American ethnicities.

### Limitations

This research is subject to four limitations. First, data are limited to California, where health care delivery may differ from the rest of the United States; therefore, our results may not be generalizable outside of California. However, California has the largest population of Asian Americans of any state,^10^ making its Asian sample the most robust in the nation. Second, the insurance variable in CHIS is hierarchical, prioritizing individuals with Medicaid or Medicare first. Therefore, those with Medicaid for part of the year and private insurance for another part were coded as having public insurance. Similarly, those without insurance for part of the year were captured in private or publicly insured categories. However, the number of individuals who changed insurance status during the year or were uninsured for only part of the year was very small and would not significantly change our results. Third, to be consistent with clinical practice guidelines, our study sample was restricted to ages 50–64, which limited our ability to identify significant differences in some Asian subgroups. For example, publicly insured South Asians had an adjusted mammography screening rate of nearly 25% points lower than publicly insured whites but, due to small numbers of publicly insured South Asians, this difference was not statistically significant. Finally, the data available in CHIS were not consistent throughout the study period and more recent data on breast and CRC screening were not available in CHIS. Despite these limitations, CHIS is the best dataset for assessing the role of insurance on preventive services by multiple Asian subgroups due to the great diversity of the Asian population in California. Furthermore, CHIS is the most accurate source of health data on Asians due to the large Asian sample size and the survey being conducted in multiple Asian languages.

## Health Equity Implications

The finding of persistent disparities in cancer screening for Asians, even for those with private insurance, highlights limitations in the ability of health insurance coverage to eliminate disparities in cancer screening among Asians. Health insurance plans and health care systems may want to review their outreach strategies to determine if they need to tailor their approach to Asian members or specific Asian subgroups with lower rates of screening. Additional research is needed to further understand why disparities in cancer screenings among privately insured Asians persist and how to eliminate these disparities. Lessons learned from the successes in the Vietnamese American community can provide valuable insights on how to increase screening rates among other subgroups of Asians.

## Abbreviations Used

ACA: Affordable Care Act
AMA: American Medical Association
CI: confidence interval
CDC: Centers for Disease Control and Prevention
CHIS: California Health Interview Survey
CRC: colorectal cancer
ER: emergency room
FOBT: fecal occult blood test
FPL: federal poverty level
N/A: not applicable
USPSTF: United States Preventive Services Task Force
VCHPP: Vietnamese Community Health Promotion Project

## Appendix

**Appendix Table A1.** Odds Ratio and 95% Confidence Intervals of Mammography, Colorectal Cancer Screening, and Flu Shot by Demographic Characteristics, Ages 50–64

	Mammography^a^ (2001–2011)	CRC screening^b^ (2001–2009)	Flu shot^c^ (2003–2011)
Race/Ethnicity-insurance type			
White-private^d^ (referent)	1.00 (1.00–1.00)	1.00 (1.00–1.00)	1.00 (1.00–1.00)
Asian-private	0.68 (0.55–0.85)	0.81 (0.71–0.92)	1.56 (1.37–1.76)
White-public^e^	0.74 (0.60–0.90)	1.00 (0.88–1.14)	1.22 (1.07–1.39)
Asian-public	0.81 (0.50–1.33)	0.76 (0.55–1.04)	2.08 (1.54–2.81)
White-uninsured^f^	0.28 (0.24–0.33)	0.36 (0.31–0.42)	0.53 (0.44–0.64)
Asian-uninsured	0.24 (0.17–0.34)	0.43 (0.33–0.56)	1.09 (0.83–1.43)
Year			
2001	0.88 (0.74–1.05)	0.58 (0.53–0.64)	N/A
2003	0.87 (0.74–1.02)	0.42 (0.38–0.46)	0.95 (0.87–1.03)
2005	1.05 (0.88–1.25)	0.58 (0.53–0.64)	0.56 (0.51–0.61)
2007	1.01 (0.84–1.21)	0.74 (0.67–0.82)	0.91 (0.84–0.99)
2009	1.07 (0.88–1.31)	1.00 (1.00–1.00)	0.95 (0.87–1.03)
2011	1.00 (1.00–1.00)	N/A	1.00 (1.00–1.00)
Gender			
Male	N/A	1.12 (1.06–1.19)	0.80 (0.76–0.85)
Female	N/A	1.00 (1.00–1.00)	1.00 (1.00–1.00)
Marital status			
Married or living as married	1.34 (1.20–1.50)	1.18 (1.11–1.25)	0.99 (0.92–1.07)
Other	1.00 (1.00–1.00)	1.00 (1.00–1.00)	1.00 (1.00–1.00)
Education			
High school or less	0.79 (0.68–0.93)	0.64 (0.59–0.71)	0.64 (0.59–0.70)
Some college	0.89 (0.77–1.02)	0.74 (0.69–0.80)	0.71 (0.65–0.77)
College graduate	0.92 (0.79–1.06)	0.86 (0.79–0.93)	0.76 (0.70–0.82)
More than college	1.00 (1.00–1.00)	1.00 (1.00–1.00)	1.00 (1.00–1.00)
Employment status			
Employed	0.87 (0.78–0.96)	0.91 (0.85–0.98)	0.85 (0.79–0.91)
Unemployed	1.00 (1.00–1.00)	1.00 (1.00–1.00)	1.00 (1.00–1.00)
English proficiency			
English proficient	0.98 (0.70–1.38)	0.99 (0.80–1.21)	0.79 (0.65–0.97)
Limited or no English proficiency	1.00 (1.00–1.00)	1.00 (1.00–1.00)	1.00 (1.00–1.00)
Percent of time in U.S.			
0–49	1.03 (0.78–1.35)	0.74 (0.64–0.87)	0.59 (0.50–0.69)
50–99	1.21 (0.97–1.50)	0.97 (0.85–1.10)	0.79 (0.71–0.89)
100	1.00 (1.00–1.00)	1.00 (1.00–1.00)	1.00 (1.00–1.00)
Income (percent of FPL)			
<100 FPL	0.67 (0.54–0.84)	0.67 (0.57–0.79)	0.76 (0.66–0.89)
100–<200 FPL	0.73 (0.63–0.85)	0.78 (0.69–0.88)	0.87 (0.77–0.99)
200+ FPL	1.00 (1.00–1.00)	1.00 (1.00–1.00)	1.00 (1.00–1.00)
Have usual place to go when sick or needing health advice			
Yes	3.01 (2.55–3.55)	2.61 (2.31–2.96)	2.43 (2.09–2.81)
No or ER is usual source	1.00 (1.00–1.00)	1.00 (1.00–1.00)	1.00 (1.00–1.00)
General health status			
Excellent/very good/good	1.15 (1.00–1.32)	0.81 (0.74–0.89)	0.69 (0.63–0.76)
Fair/poor	1.00 (1.00–1.00)	1.00 (1.00–1.00)	1.00 (1.00–1.00)
Smoking status			
Current smoker	0.60 (0.53–0.69)	0.65 (0.60–0.71)	0.74 (0.67–0.81)
Current nonsmoker	1.00 (1.00–1.00)	1.00 (1.00–1.00)	1.00 (1.00–1.00)
Age	1.02 (1.01–1.04)	1.10 (1.09–1.11)	1.06 (1.05–1.07)

Source: CHIS 2001, 2003, 2005, 2007, 2009, 2011/2012.

^a^ Mammogram within the past 2 years.

^b^ FOBT in the past year, sigmoidoscopy in the past 5 years, or colonoscopy within the past 10 years.

^c^ Flu shot within the past year.

^d^ Private insurance includes insurance purchased by individuals or employers.

^e^ Public insurance includes Medicare, Medicaid, and other forms of public coverage such as military.

^f^ Anyone without public or private insurance was classified as uninsured.

CHIS, California Health Interview Survey; CRC, colorectal cancer; ER, emergency room; FOBT, fecal occult blood test; FPL, federal poverty level; N/A, not applicable.

**Appendix Table A2.** Odds Ratio and 95% Confidence Intervals of Mammography, Colorectal Cancer Screening, and Flu Shot by Demographic Characteristics, Ages 50–64

	Mammography^a^ (2001–2011)	CRC Screening^b^ (2001–2009)	Flu Shot^c^ (2003–2011)
Race/Ethnicity-insurance type			
White-private^d^ (referent)	1.00 (1.00–1.00)	1.00 (1.00–1.00)	1.00 (1.00–1.00)
Chinese-private	0.56 (0.41–0.76)	0.95 (0.79–1.14)	1.41 (1.18–1.67)
Filipino-private	0.77 (0.50–1.19)	0.69 (0.53–0.90)	1.68 (1.30–2.18)
Japanese-private	0.83 (0.55–1.25)	0.82 (0.64–1.06)	1.52 (1.19–1.94)
Korean-private	0.42 (0.24–0.72)	0.72 (0.52–0.99)	2.00 (1.43–2.81)
South Asian-private	0.62 (0.37–1.04)	0.67 (0.45–0.98)	1.38 (1.03–1.85)
Vietnamese-private	1.10 (0.53–2.29)	1.30 (0.93–1.82)	2.30 (1.61–3.30)
White-public^e^	0.76 (0.62–0.93)	1.01 (0.88–1.15)	1.24 (1.09–1.41)
Chinese-public	0.59 (0.20–1.71)	0.68 (0.36–1.29)	1.73 (0.95–3.16)
Filipino-public	0.79 (0.30–2.04)	1.66 (0.86–3.19)	1.65 (0.89–3.08)
Japanese-public	0.28 (0.04–1.78)	0.28 (0.07–1.13)	1.38 (0.45–4.27)
Korean-public	0.66 (0.24–1.83)	0.39 (0.17–0.90)	4.87 (2.17–10.94)
South Asian-public	0.19 (0.02–1.68)	0.29 (0.04–2.45)	0.58 (0.14–2.46)
Vietnamese-public	1.13 (0.54–2.35)	0.87 (0.58–1.29)	2.94 (1.92–4.52)
White-uninsured^f^	0.28 (0.24–0.34)	0.36 (0.31–0.42)	0.53 (0.44–0.64)
Chinese-uninsured	0.24 (0.14–0.42)	0.30 (0.19–0.46)	0.84 (0.55–1.28)
Filipino-uninsured	0.23 (0.10–0.54)	0.46 (0.26–0.83)	1.16 (0.50–2.66)
Japanese-uninsured	0.08 (0.03–0.19)	0.31 (0.11–0.86)	0.55 (0.18–1.68)
Korean-uninsured	0.16 (0.09–0.26)	0.31 (0.20–0.49)	1.47 (0.95–2.27)
South Asian-uninsured	0.27 (0.05–1.33)	0.29 (0.08–1.12)	0.93 (0.25–3.43)
Vietnamese-uninsured	0.57 (0.31–1.04)	1.06 (0.63–1.79)	2.00 (1.27–3.15)
Year			
2001	0.87 (0.73–1.04)	0.58 (0.53–0.64)	N/A
2003	0.86 (0.73–1.01)	0.42 (0.38–0.46)	0.94 (0.87–1.03)
2005	1.04 (0.87–1.24)	0.58 (0.53–0.64)	0.56 (0.51–0.61)
2007	1.00 (0.84–1.19)	0.74 (0.67–0.82)	0.91 (0.84–0.98)
2009	1.06 (0.87–1.28)	1.00 (1.00–1.00)	0.95 (0.87–1.03)
2011	1.00 (1.00–1.00)	N/A	1.00 (1.00–1.00)
Gender			
Male	N/A	1.12 (1.06–1.19)	0.80 (0.76–0.86)
Female	N/A	1.00 (1.00–1.00)	1.00 (1.00–1.00)
Marital status			
Married or living as married	1.34 (1.20–1.50)	1.18 (1.11–1.25)	0.99 (0.92–1.07)
Other	1.00 (1.00–1.00)	1.00 (1.00–1.00)	1.00 (1.00–1.00)
Education			
High school or less	0.78 (0.67–0.91)	0.64 (0.58–0.70)	0.64 (0.59–0.69)
Some college	0.88 (0.77–1.01)	0.74 (0.69–0.80)	0.70 (0.65–0.76)
College graduate	0.92 (0.80–1.07)	0.87 (0.80–0.94)	0.76 (0.70–0.82)
More than college	1.00 (1.00–1.00)	1.00 (1.00–1.00)	1.00 (1.00–1.00)
Employment status			
Employed	0.86 (0.78–0.96)	0.92 (0.85–0.98)	0.85 (0.79–0.91)
Unemployed	1.00 (1.00–1.00)	1.00 (1.00–1.00)	1.00 (1.00–1.00)
English proficiency			
English proficient	0.96 (0.67–1.37)	1.09 (0.88–1.35)	0.89 (0.72–1.11)
Limited or no English proficiency	1.00 (1.00–1.00)	1.00 (1.00–1.00)	1.00 (1.00–1.00)
Percent of time in U.S.			
0–49	1.01 (0.76–1.33)	0.76 (0.65–0.89)	0.59 (0.50–0.70)
50–99	1.22 (0.98–1.54)	0.97 (0.85–1.11)	0.78 (0.69–0.88)
100	1.00 (1.00–1.00)	1.00 (1.00–1.00)	1.00 (1.00–1.00)
Income (percent of FPL)			
<100 FPL	0.64 (0.52–0.80)	0.67 (0.56–0.79)	0.76 (0.65–0.88)
100–<200 FPL	0.71 (0.62–0.83)	0.77 (0.69–0.87)	0.87 (0.77–0.98)
200+ FPL	1.00 (1.00–1.00)	1.00 (1.00–1.00)	1.00 (1.00–1.00)
Have usual place to go when sick or needing health advice			
Yes	2.98 (2.52–3.52)	2.60 (2.31–2.94)	2.45 (2.12–2.84)
No or ER is usual source	1.00 (1.00–1.00)	1.00 (1.00–1.00)	1.00 (1.00–1.00)
General health status			
Excellent/very good/good	1.17 (1.02–1.35)	0.82 (0.74–0.89)	0.71 (0.64–0.77)
Fair/poor	1.00 (1.00–1.00)	1.00 (1.00–1.00)	1.00 (1.00–1.00)
Smoking status			
Current smoker	0.61 (0.53–0.70)	0.65 (0.60–0.71)	0.74 (0.68–0.81)
Current nonsmoker	1.00 (1.00–1.00)	1.00 (1.00–1.00)	1.00 (1.00–1.00)
Age	1.02 (1.01–1.04)	1.10 (1.09–1.11)	1.06 (1.05–1.07)

Source: CHIS 2001, 2003, 2005, 2007, 2009, 2011/2012.

^a^ Mammogram within the past 2 years.

^b^ FOBT in past year, sigmoidoscopy in past 5 years, or colonoscopy within the past 10 years.

^c^ Flu shot within the past year.

^d^ Private insurance includes insurance purchased by individuals or employers.

^e^ Public insurance includes Medicare, Medicaid, and other forms of public coverage such as military.

^f^ Anyone without public or private insurance was classified as uninsured.
